# Formins: Emerging Players in the Dynamic Plant Cell Cortex

**DOI:** 10.6064/2012/712605

**Published:** 2012-09-26

**Authors:** Fatima Cvrčková

**Affiliations:** Department of Experimental Plant Biology, Faculty of Science, Charles University, Viničná 5, 128 43 Prague, Czech Republic

## Abstract

Formins (FH2 proteins) are an evolutionarily conserved family of eukaryotic proteins, sharing the common FH2 domain. While they have been, until recently, understood mainly as actin nucleators, formins are also engaged in various additional aspects of cytoskeletal organization and signaling, including, but not limited to, the crosstalk between the actin and microtubule networks. A surprising diversity of domain organizations has been discovered among the FH2 proteins, and specific domain setups have been found in plants. Seed plants have two clades of formins, one of them (Class I) containing mostly transmembrane proteins, while members of the other one (Class II) may be anchored to membranes via a putative membrane-binding domain related to the PTEN antioncogene. Thus, plant formins present good candidates for possible mediators of coordination of the cortical actin and microtubule cytoskeletons, as well as their attachment to the plasma membrane, that is, aspects of cell cortex organization likely to be important for cell and tissue morphogenesis. Although experimental studies of plant formin function are hampered by the large number of formin genes and their functional redundancy, recent experimental work has already resulted in some remarkable insights into the function of FH2 proteins in plants.

## 1. Cortical Cytoskeleton in Plant Cell Growth, Morphogenesis and Differentiation

Plants possess two cytoskeletal systems shared by all eukaryotes, that is, the actin filament and microtubule networks, with a host of associated and regulatory proteins. Cytoskeletal networks in the cell cortex are crucial for the controlled remodeling of the plant cell wall, contributing thus substantially to cell growth and morphogenesis. In particular, polar cell growth, including both tip growth (encountered, e.g., in root hairs and pollen tubes) and nonisodiametric cell expansion (occurring as one-dimensional elongation, e.g., in the root elongation zone, as two-dimensional expansion e.g., in epidermal cells, or as localized expansion, e.g., during *Arabidopsis* trichome differentiation or shaping of epidermal pavement cell lobes) involves intricate co-ordination of cytoskeletal remodeling and membrane turnover (see e.g., [1, 2]). Polarized exocytosis, directed and/or restricted by cytoskeletal structures, may also take place without an increase in cell size, as documented by localized deposition of molecules, in particular proteins, to distinct regions of the cell cortex or plasmalemma (comparable perhaps to the notorious example of apical versus basolateral polarity of metazoan epithelia). The same cell may exhibit several of these phenomena in the course of its life or simultaneously. For example, many cells deposit distinct proteins, such as, for example, PIN family auxin carriers, to their crosswalls while elongating (see [3–6]). Last but not least, cytokinesis, or cell division, may be viewed as a special case of “inward-oriented” cell growth with new cell wall material deposition oriented towards a specific intracellular compartment, the nascent cell plate.

Most attention has been so far devoted to cortical microtubules, which delimit areas where new cell wall material is inserted (reviewed in [7]). Local randomization of the cortical microtubule network is among the first observable events during transition from polar expansion to tip growth in lettuce trichoblasts [8], although this may be species specific, as no such phenomenon was observed in alfalfa (*Medicago*) [9]. Cortical miocrotubules were long believed to determine the direction of cellulose microfibrils of the primary cell wall, and therefore also of cell expansion, in elongating cells. However, their disruption by the microtubule-depolymerizing herbicide oryzalin [10], or by the temperature-sensitive *mor1-1* mutation, which affects a microtubule-associated protein, results in loss of polarity and cell swelling while microfibrils remain ordered [11, 12]. Thus, microfibrils can organize in a microtubule-independent fashion, possibly by self-assembly driven by physical forces, while the role of microtubules may be, at least in some cases, restricted to determining microfibril length (see [7, 13]). 

In plant tip-growing cells microtubules may control growth direction rather than growth itself, as microtubule disruption by oryzalin results in wavy root hair growth in *Arabidopsis* (see [14]), and depletion of tubulin by antisense RNA even induces ectopic root hair formation, branching, and occasional initiation of multiple hairs per bulge [15]. Also, in poppy pollen tubes, microtubules seem to be important for maintaining growth direction but not for growth as such [16], and extremely high concentrations of the microtubule-stabilizing drug taxol were required to inhibit tobacco pollen tube elongation [17].

It is becoming increasingly obvious that actin also plays an important part in plant cell morphogenesis, including polarized cell expansion. Specific *Arabidopsis* actin isoforms have been reported to participate in tip growth of root hairs, or at least to be abundantly expressed in tip-growing cell types [18–21], while others take part in diffuse cell growth during root elongation or callus expansion [19, 22, 23]. Interaction with actin may also contribute to the role of microtubules in expanding cells. In the thermosensitive *Arabidopsis* mutant *rsw6*, cortical microtubules are aligned within a cell but their position with respect to the root axis randomizes at the restrictive temperature, resulting in root swelling that can be prevented by LatB-induced actin depolymerization [24]. Moreover, LatB elicits swelling of *Arabidopsis* rhizodermis cells at high doses, and aggravates the effects of the *mor1-1* mutation at low concentration, indicating a crosstalk between the two cytoskeletal systems [25].

*Arabidopsis* mutants in genes of the *DISTORTED* (*DIS*) class exhibit a syndrome that phenocopies the effects of anti-actin drugs LatB or CytD, characterized by deformed trichomes and misshapen epidermal pavement cells. This is accompanied by microtubule disorganization, and possibly stabilization, apparently secondary to disruption of actin [26, 27]. Four of the *DIS* genes code for subunits of the Arp2/3 actin nucleation complex [27–31]. Surprisingly, *Physcomitrella* mutants deficient in Arp2/3 subunits exhibit partial loss of cell polarity but also reduced tip growth, suggesting that the relatively insignificant role of this complex in tip growth may be specific to angiosperms [32, 33]. However, as we shall see below, in seed plants, Arp2/3 is apparently not the only actin-nucleating complex involved in localized cell expansion, albeit fine branched filaments nucleated by Arp2/3 are present in the cortex of diffusely growing tissue culture cells [34].

In tip-growing root hairs, actin filaments participate in root hair emergence at the bulge stage, and later form a fine dynamic network in the extending tip, merging into thicker cables in older parts of the hair. As the hair matures and ceases growing, cables extend into the area previously occupied by the fine meshwork, suggesting a causal connection between tip growth and the presence of fine actin arrays [35–38]. Reversible disruption of the actin cytoskeleton by mild doses of inhibitors (LatB, CytD), sufficient to destroy the fine meshwork but not actin bundles, causes temporary tip swelling, either by mislocalized exocytosis or by perturbation of endocytosis. The former is more likely, as the pattern of internalization of the FM4-64 dye, which can serve as an indicator of endocytosis, does not change in root hairs temporarily depolarized by CytD [36]. At least part of the actin function in root hairs is myosin dependent, as documented by reduced root hair growth in *Arabidopsis* lacking one of the myosin XI isoforms [39]. 

**Figure 1:**
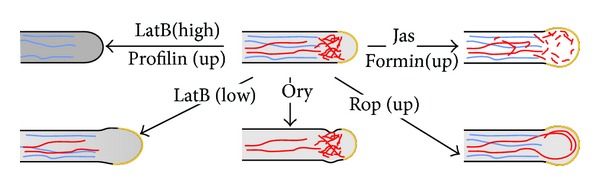
Schematic representation of the effects of perturbations of the actin (red) or tubulin (blue) networks in a tip-growing plant cell by pharmacological treatments or mutations, which reveal distinct roles for thick actin cables, fine cortical actin network, and microtubules. Ory: oryzalin, LatB: latrunculin B, and Jas: jasplakinolide. Growing portion of the cell surface is shown in yellow; shading indicates the levels of monomeric G-actin.

Actin organization in pollen tubes resembles that in root hairs [40–42], with bundles of parallel filaments along the shank of the tube and a fine meshwork of randomly oriented short filaments in the tip region [43]. Like root hairs, pollen tubes of both angio- and gymnosperms respond to mild LatB treatment by tip swelling [44, 45]. However, swelling was observed also in lily pollen tubes treated by the actin-stabilizing drug jasplakinolide, which induced formation of short, thick actin cables throughout the tip region [46]. The effects of various cytoskeleton-perturbing treatments on tip-growing plant cells are summarized in Figure 1. 

Localized secretion in nonexpanding cells also relies on cytoskeletal cues. Microtubules determine the position of secretory domains in *Arabidopsis* seed coat cells that deposit pectinaceous mucilage into their periplasm [47]. Also the localization of the PIN1 auxin efflux carrier at bottom-oriented crosswalls of root cortex cells is established in a microtubule-dependent manner already in the course of cytokinesis, while actin may be involved rather in endocytotic turnover of PIN auxin transporters [3, 4]. Localization of the AUX1 auxin influx carrier to the opposite cell poles is also actin-dependent [48]. Bundling of actin elicited by expression of a mouse talin-derived YFP fusion protein in cultured tobacco cells resulted in changes in cell division pattern consistent with perturbation of auxin transport, possibly due to altered localization of auxin transporters [49].

Cytokinesis in plant cells is intimately linked to cytoskeletal rearrangements as well. Prior to onset of mitosis, microtubules form a preprophase band that determines the position of the future cell plate (reviewed in [7]). Vigorous membrane turnover takes place at the adjacent plasmalemma, possibly associated with deposition of local markers that determine the future sites of cell plate fusion [50, 51]. After anaphase, microtubules reorganize into the phragmoplast that acts as scaffolding for assembly of the nascent cell plate by vesicle fusion. One of the alleles of the above discussed *MOR1* gene was previously identified as *gem1* based on a pollen cytokinesis defect [52]; detailed analyses revealed occasional occurrence of multinucleate cells and misaligned cell plates even in the *mor1-1* mutant originally believed to suffer only from a cell polarity defect [53, 54]. Microtubules, microfilaments and associated molecular motors (kinesins and myosins) all participate in phragmoplast function and cell plate formation [55–57].

The complex dynamics of the cortical cytoskeleton—closely interlinked with that of the plasmalemma, underlying cortical cytoplasm and the endomembrane system—is orchestrated by an intricate regulatory network including, among others, lipid-based and redox-based signalling pathways, as well as small GTPases of the Rop (Rho of plants) family with their co-factors (reviewed, e.g., in [6, 58–60]). 

Given the many interlinked roles of the cytoskeletal networks in plant cell growth and cell morphogenesis, any protein affecting cytoskeletal function is likely to influence also these processes, and formins, a major family of evolutionarily conserved actin nucleators with a host of possible additional functions, present in most, if not all eukaryotes [61], should be no exception. 

## 2. Formins as Cytoskeletal Organizers and Signaling Hubs

Formins, or FH2 proteins, are a large, ancient family of eukaryotic proteins sharing the evolutionarily conserved FH2 (formin homology 2) domain, usually, though not always, located in the C-terminal portion of the protein and preceded by a proline-rich FH1 domain [62–66]. The first member of the family was originally described on the basis of a mouse insertional mutation responsible for a limb deformity defect [67], and homologs were later found across vertebrates and yeasts [68–74]. Somewhat ironically, the originally described limb phenotype, which even rose to a brief fame of one of “poster cases” documenting that transgenosis as such may negatively affect animal welfare [75], turned out to be due not to disruption of the formin gene but to perturbed expression of a neighboring gene, gremlin, whose regulatory sequences overlap the formin locus [76]. While formin itself may also contribute to the limb phenotype through possible participation in modulating the secretion of extracellular regulatory peptides [77], it is noteworthy that involvement of formin genes in genome regions subjected to “large-scale” regulation involving multiple genes is, in at least one case, found also in plants (see discussion of AtFH5 below).

Until the end of the last millennium, yeast and metazoan FH2 proteins have been implicated in numerous cellular processes, often related to cytoskeletal, in particular microtubule-related activities (e.g., mitosis [74], cytoplasmic streaming [78], or cytokinesis [79]). However, they have been also localized the to cell nucleus [80, 81] and implicated in several signaling pathways whose ultimate outcome is the regulation of gene expression [82]. 

Metazoan FH2 proteins form a large gene family that can be classified into several distinct subfamilies [70, 83–85]. Among them, members of the best characterized Diaphanous clade (Diaphanous-related formins, or DRFs) exhibit a characteristic domain structure that is found also in fungal and *Dictyostelium* formins and thus believed to be ancient [86]. The DRFs contain a regulatory N-terminal region (GBD, sometimes also termed FH3 [87]) that can alternatively bind either to an autoinhibitory domain (DID) at the proteins' C-end or to an activated (GTP-bound) small GTPase of the Rho clade, leading to Rho-dependent release of the autoinhibitory interaction and thereby to formin activation [88–90]. Rho-related small G-proteins, such as Rho *sensu stricto*, Rac and Cdc42 (Cft) of metazoans and fungi, or Rop of plants, are notorious for their role in the control of cell polarity, predominantly via regulating the actin cytoskeleton (reviewed e.g., in [61, 91–94]). However, not all functions of Diaphanous-like formins are exclusively actin-related; for instance, mammalian homologs localize to the mitotic spindle and contribute to the actin-microtubule crosstalk [95, 96]. As a rule, formins harbor the hallmark FH2 domain at or close to their C-end, preceded usually by the FH1 domain and by additional domains mediating regulatory interactions; for instance, the well-known DRFs have an additional conserved motif located C-terminally from the FH1/FH2 tandem [83, 84, 86, 97]. A remarkable exception from the usual domain order are the metazoan “inverted formins” (INFs) with the FH1 and FH2 domains located N-terminally and followed by a large C-terminal extension [98].

A decisive turn in the quest for the molecular mechanism of formin action was the discovery of an actin-nucleating ability in yeast formins [99, 100]. Subsequently, formins were recognized as a new class of actin nucleators, functioning by a mechanism independent of the Arp2/3 complex. Unlike the Arp2/3 complex, which associates with the pointed end of actin filaments, a dimer of formin's FH2 domains acts as a processive or “leaky” cap at the barbed ends ([101]; for a review see, e.g., [64, 102–104]). While this cap may, at least for some formins, facilitate polymerization of G-actin subunits (whose supply is aided by the FH1 domain that acts as a “docking site” for profilin-actin complexes), other formins have been documented to function as “mere” capping proteins not engaged in actin polymerization (e.g., in the case of the fission of yeast cdc12 protein, which acts a profilin-gated cap at the barbed end of actin filaments—[105]). The dimerization ability of FH2 domains raises also the question whether, and with what functional consequences, could various members of the extensive formin family form heterodimers. Surprisingly little is known about this topic, and heterodimerization, (moreover mediated not by FH2 but by the GBD and DID motifs), was so far well documented only among closely related mammalian DRFs and between DRFs and INFs [106, 107]. 

Besides direct actin nucleation, some formins may participate in the initiation of new actin filaments also indirectly, in particular via communication with other nucleation complexes. Spire, a metazoan actin-nucleating protein that is often associated with endosomal membranes, is known to interact directly with several formins (see [108]). Co-ordination between formins and the Arp2/3 complex may be mediated by common interactors, such as the IQGAP class of Rho GTPase activators that can, besides their function as G-protein cofactors, also directly bind both to the Arp2/3 activator WASP homologs and to Diaphanous-related formins in metazoans [109]. In *Drosophila*, the WASP family protein Wash mediates Rho-based regulation of both Arp2/3-dependent and Spire/formin-dependent actin nucleation [110]. Based on yeast and metazoan data, formins have been suggested to stimulate formation of actin bundles, while “classical” Arp2/3-driven actin nucleation promotes establishment of fine branched filament arrays [63]. Indeed, some formins, including the *Arabidopsis thaliana* AtFH1 protein (see below) are capable of bundling actin filaments side by side and/or crosslinking them [111–115]. 

In particular in the recent years, numerous reports suggesting that formins participate more or less directly in the organization of the yeast and metazoan microtubular cytoskeleton have appeared [96, 98, 116–119]; in several of these cases, direct binding between formins (in particular DRFs and INFs) and microtubules has been documented. Similar observations have been made also in plants ([120–123]; see also below). Some formins may be also involved in the coordination of the actin and intermediate filament systems (e.g., [124]).

While formins thus emerge as major regulators of diverse cytoskeletal functions, at least some of them may participate also in additional cellular processes less directly related to the cytoskeleton or cell morphogenesis, in particular in the nuclear events associated with gene expression. The *Caenorhabditis fozi-1* gene encodes a protein that contains both the FH2 domain and a DNA-binding zinc finger domain, and acts as a transcriptional regulator [125]. A family of formin-binding proteins (FBPs) participates in spliceosome assembly, raising the possibility that formins may be engaged also in pre-mRNA processing [126].

To summarize: besides some additional functions restricted only to certain members of the extensive gene family, FH2 proteins appear to play a part in numerous cytoskeleton-related processes, including those commonly occurring in the eukaryotic cell cortex. Indeed, cortical localization and/or association with membranes, including (but not limited to) the plasmalemma, has been documented for numerous paralogs in both yeasts and metazoans (see, e.g., [116, 127–131]), and formins have been implicated in the development of cortical structures such as cell-to-cell junctions [131] or filopodia [132, 133] in metazoan cells. It can therefore be expected that formins might have analogous roles in cell cortex organization also in plant cells, whose rigid cell walls make the precise coordination between the cortical cytoskeleton and the endomembrane system even more important. 

## 3. Formins in Plants: New Uses for an Old Domain

The first reports of FH2 proteins in plants date from 2000, when an *Arabidopsis thaliana* formin gene, AFH1 (later renamed AtFH1) has been cloned, and its product found to associate with membranes due to the presence of a N-terminal membrane insertion signal and a single transmembrane helix, features found also in additional putative genes predicted from the *Arabidopsis* genome sequence [134, 135]. After completion of *A. thaliana* genome sequencing [136], it became obvious that plant FH2 proteins form an extensive family (e.g., *Arabidopsis* has 21 paralogs, some of them possibly encoding multiple protein isoforms due to alternative splicing) with two distinct subfamilies, termed Class I and Class II, in flowering plants [104, 137, 138] (note that the current terminology of *Arabidopsis* formin genes was established in [138]). A third plant formin clade (Class III), related to Class I, has been found only in nonangiosperm “lower” plants such as the moss *Physcomitrella patens*, the lycophyte *Selaginella moelendorffii,* and some algae [84].

**Figure 2:**
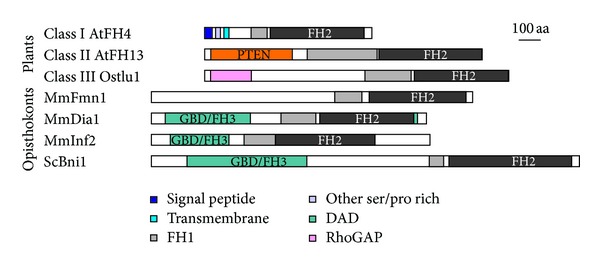
Domain layout of typical representatives of selected formin families from plants and opisthokonts (yeasts and metazoans). At—*Arabidopsis thaliana*, Ostlu : *Ostreococcus lucimarinus* (a prasinophyte alga), Mm : *Mus musculus*, and Sc : *Saccharomyces cerevisiae*. Modified from [83], for sequence details and accessions see there.

Each plant formin clade exhibits a characteristic domain layout (Figure 2;  [84, 138]), although exceptions are common, reflecting possibly rapid evolution of multigene families with a great deal of degeneracy (“redundancy”) and resulting relaxation of evolutionary constraints. Indeed, especially within Class II, orthologs cannot be clearly assigned even between *A. thaliana* and *A. lyrata*, two closely related species that have separated mere five million years ago, while Class I appears to be better conserved. Nevertheless, no evidence of positive selection, that is, selection favoring diversification, was found in the FH2 domains of Class II formins, albeit it is still possible that positive selection operated on the N- and C-terminal portions of the proteins [139]. 

A typical representative of the angiosperm Class I formins (represented by 11 genes, AtFH1 to AtFH11, in *Arabidopsis*) has an N-terminal membrane insertion signal sequence, followed by a presumably extracellular proline-rich domain containing motifs reminiscent of some cell wall (glyco)proteins including extensins, known to participate in cell wall loosening, which is a prerequisite of cell growth [38, 140]. Downstream of these extracytoplasmic motifs is a single amphipathic transmembrane helix followed by a cytoplasmic portion with C-terminally located FH1 and FH2 domains. Parts of the protein between the transmembrane segment and the FH1-FH2 tandem are loosely conserved among some formin isoforms (see also [120] and the discussion of the GOE motif below). 

Class II formins (represented by AtFH12 to AtFH21 in *Arabidopsis*) lack membrane insertion signals and are supposed to be exclusively cytoplasmic. Their N-terminal portion usually contains a domain related to the mammalian antioncogene PTEN (phosphatase and tensin homolog on chromosome ten, [141–143]). This domain is generally considered a lipid and protein phosphatase, acting preferentially on membrane phospholipids [141, 144]. However, while plants do have genuine enzymatically active PTEN homologs [145], the PTEN-like domain of plant Class II formins lacks critically important conserved arginine residue forming part of the phosphatase active site. This makes enzymatic activity extremely unlikely, and Class II PTEN-like domains have been proposed to mediate binding of the FH2 proteins to membranes [138]. 

Remarkably, Class III formins also carry a conserved domain that appears to have lost its original function due to a point mutation, namely, a RhoGAP-like domain, homologous to the GTPase-activating proteins associated with Rho clade small GTPases, but missing a critical conserved arginine finger motif. While this domain may have retained its ability to bind small GTPases, it is rather unlikely to be a genuine GTPase activator (unless it employs some alternative mechanism). Plant Class III formins would thus share the ability to bind Rho family members, common among GBD/FH3- carrying FH2 proteins such as the DRFs or fungal formins. The phylogenetic distribution of domain architectures among plant formins is compatible with a relatively simple evolutionary scenario where PTEN-like domains of Class II formins and RhoGAP-like domains (characteristic for Class III) have replaced the ancestral GBD/FH3 domains early in the plant lineage while maintaining a continuity of the Rho-FH2 and/or membrane-FH2 association. The subsequent acquisition of transmembrane domains in some formins (i.e., Class I), conspicuously coinciding with expansion of the formin family and also with the ascent of plants to dry land, was finally followed by loss of Class III formins in the lineage leading to angiosperms [84].

While all plant FH2 domains detected so far in genome databases can be unambiguously assigned to one of the three above-described classes (or only two classes in case of angiosperms), not all of them conform to the domain architecture typical for their class. Even in angiosperms, several Class I proteins (e.g., *Arabidopsis* AtFH7) lack the N-terminal membrane insertion sequences, and quite many Class II formins deviate from the canonical PTEN-FH1-FH2 domain order. Out of the ten *A. thaliana *Class II formins, only four, AtFH13, AtFH14, AtFH18, and AtFH20, exhibit the canonical domain configuration [138]. However, aside of (non-FH2) domain losses and occasional internal duplications, plant formins as a rule do not contain additional conserved sequence motifs, with one remarkable exception of a *Physcomitrella patens* gene encoding a protein with a N-terminal Sec10-related domain and C-terminal FH1/FH2 domains [84, 146]. Sec10 is one of the eight subunits of the evolutionarily conserved exocyst complex responsible for exocytotic vesicle addressing towards distinct plasmalemma domains (but sometimes also other targets, such as, e.g., the nascent cell plate); its function is thus intimately interlinked with that of other cortical structures, including the cytoskeleton (see e.g., [6, 58, 147–149]). A direct link between a formin and a subunit of the exocyst may therefore be biologically meaningful. It is, however, not yet clear whether the suspected Sec10-formin fusion protein is expressed *in vivo* in the moss, or if the locus merely encodes alternative gene products whose expression may perhaps be somehow coordinated [146].

The large number of formin isoforms encoded by plant genomes raises several intriguing questions. First of all, it is far from clear how many distinct formin dimers can exist in plant cells; while very little is known about heterodimerization ability of formins (see above), this possibility cannot be excluded. Were the FH2 domains capable of free mutual interactions (and were they all coexpressed, which is obviously not the case), *Arabidopsis* would be able to generate up to 484 binary FH2 domain combinations (taking into account the presumed two distinct gene products for AtFH15 [138]). While the actual biologically relevant number is likely to be much lower, even if formin-formin interactions were restricted to homodimerization, there would still be at least 21 possible functional formin complexes. Since duplicated genes of identical function are likely to be eliminated by natural selection unless contributing to the fitness of the organism [150], surviving numerous formin varieties can be expected to differ functionally. Besides genuine functional specialization (subfunctionalization or neofunctionalization—[151]), in sessile organisms, such as plants (and also yeasts—see [152]), the evolutionary advantage may be provided by “fine-tuning” that optimizes the protein function for specific intracellular or intraorganismal locations or environmental conditions.

Last but not least, “redundancy” (or degeneracy) within the plant formin family is also a practical problem for researchers interested in the function of these proteins, since loss-of-function mutations can be, to an extent, compensated for by unaffected members of the gene family. This has also significantly hampered experimental studies of plant formins, making the gene family perhaps less attractive for researchers than it would deserve.

## 4. Functional Studies of Plant Formins

The majority of experimental work devoted to plant FH2 proteins has focused on members of the angiosperm Class I. This clade includes AtFH1, the first plant formin to be cloned [134], and also the most ubiquitously expressed (and thus presumably housekeeping) member of the formin family in *Arabidopsis thaliana* vegetative tissues according to publicly available microarray data [153]. The predicted membrane localization of AtFH1 was confirmed; this formin was also found to interact with FIP2 [134], a protein originally predicted to interact also with potassium membrane channels but later discovered to encode a putative E3 ubiquitin ligase [154]. Recently, it was demonstrated that AtFH1 preferentially localizes to membrane regions not occupied by microtubules, and that actin bundling elicited by AtFH1 overexpression depends on anchoring the formin within the cell wall and results in decreased organelle motility [155]. 

Also other members of the Class I formin family were found in the plasmalemma. Two closely related *Arabidopsis* formins from the Class I branch known as group Ie, AtFH4 and AtFH8, localize preferentially to plasmalemma adjacent to transversal cell walls in the rhizodermis cell files [156], that is, to actin-rich domains of the cell surface that exhibit intensive vesicle trafficking [157] and that are specifically enriched with auxin transporters. AtFH5 is localized in the cell plate in the course of cytokinesis [158]. AtFH6, another Class I formin, which is massively upregulated in expanding giant cells of nematode-induced galls, localizes to the cytoplasmic membrane in these cells [159]. Fluorescent protein-tagged AtFH6 was found at or around the nascent cell plate and newly developed cross-walls both in *Arabidopsis* seedlings and when heterologously expressed in cultured tobacco BY-2 cells [160].

Heterologous high-level expression of AtFH1 in tobacco pollen tubes [161] caused tube tip swelling with excessive formation of actin cables (see also Figure 1). Actin-bundling activity of AtFH1 [111, 112] might have contributed to the observed phenotype. Since AtFH1 is normally not expressed in pollen under normal circumstances, possible artifacts due to ectopic overexpression also cannot be excluded. However, overexpression of other plant formins such as AtFH8, another *Arabidopsis* formin that is normally expressed in root tissues and apparently lacks the bundling activity, elicits formation of abundant actin cables, partial depolarization and branching in *Arabidopsis* root hairs [162], while expression of a nonfunctional derivative of AtFH8 suppresses root hair growth [156]. Even more remarkably, a heterologous (but not ectopic) overexpression of AtFH3, the main *Arabidopsis* Class I pollen formin, in tobacco pollen tubes also caused extensive actin bundling, followed by tube tip swelling, while inhibition of AtFH3 expression in *Arabidopsis* by RNAi led to partial inhibition of pollen tube growth [163]. Manipulation of the expression levels of closely related AtFH5, which is also expressed in pollen, and its tobacco homolog NtFH5, could shift the balance between longitudinal actin cables and the fine apical meshwork, with subsequent alterations in tube growth rate. In addition, inhibition of NtFH5 by RNAi often resulted in wavy pollen tube growth [164], suggesting that also microtubules might perhaps be compromised. 

These observations are all consistent with a general requirement for finely balanced Class I formin-based actin nucleation in tip-growing cells of higher plants. Remarkably, disruption of the Arp2/3 actin nucleation pathway has only subtle effects in tip-growing cells while profoundly affecting other modes of cell expansion (see [1, 165] and above). However, in the moss *Physcomitrella patens*, Class I formins are dispensable for tip growth as such, since simultaneous RNAi silencing of all six moss Class I members only led to the reduction in cell growth and division, that is, “mere” quantitative growth defects without obvious morphogenetic phenotypes, in contrast to Class II formins which were found to be indispensable for maintaining cell polarity ([166], see also below).

Several plant Class I formins, including *Arabidopsis* AtFH1 [111], AtFH3 [163], AtFH4 [156], AtFH5 [158] and AtFH8 [162], have been demonstrated to nucleate actin* in vitro*. However, similar to their metazoan counterparts, plant Class I formins may have additional roles besides those directly related to actin organization and membrane anchoring. In particular, the Group Ie formin AtFH4 was demonstrated to bind microtubules both *in vitro* and *in vivo* via a conserved sequence motif that is characteristic for the Ie branch of Class I, the GOE domain, which is identical with the mutually related plant-specific domains of unknown function previously annotated as ProDom PD038281 and PD224441. AtFH4 also appears to be capable to mediate alignment of the endoplasmic reticulum along microtubules under certain circumstances [120].

As mentioned above, the use of mutants in plant formin studies is complicated by the degeneracy of the extensive formin family. Indeed, very few phenotypes were described for plant FH2 protein mutants, even in case of Class I that, as a rule, contains relatively abundantly expressed proteins. Loss of AtFH5 leads to a cytokinesis delay during endosperm cellularization, consistent with cell plate localization of the AtFH5 protein; the tissue-specific defect is well explained by high levels of AtFH5 expression in the endosperm, and thus possibly its increased functional importance [158]. While the subsequent recovery of cytokinesis in AtFH5 mutants may be due to compensation by some other member(s) of the large formin family [137, 138], it is worth noting that in tobacco BY2 cells the actin polymerization inhibitor bistheonellide A caused only a temporary delay of cytokinesis but not its permanent disruption; later stages of cell plate development, where major involvement of endocytosis is suspected, were affected more profoundly [57]. It is tempting to speculate that the relative importance of microfilaments and microtubules in cytokinesis may be opposite to that described for tip growth. 

To complicate matters further, the *Arabidopsis* AtFH5 gene is involved in rather complex transcriptional regulation involving extensive chromatin modifications, somewhat reminiscent to the mammalian formin/gremlin gene tandem discussed above. Its expression is controlled by Polycomb-group proteins and the gene itself is maternally imprinted [167]. In our hands, young (less than two weeks old) seedlings of the reporter line used to characterize the endosperm-specific expression pattern [158] exhibited gene expression only in various root tissues and cotyledon vasculature. However, gene expression patterns in other tissues were highly variable and often changed in the course of development. In particular, true leaves exhibited only rare patches of gene expression in the vascular bundles, and even in the root tips gradual decline of reporter gene expression was observed (F. C. and Ann Sibyl Kuckuck, unpublished observations).

While loss of function mutants in genes from large, degenerate families are generally devoid of observable phenotypes due to compensation by their sibling loci, combination with mutations and/or pharmacological treatments affecting their targets (such as asymptomatic doses of cytoskeletal inhibitors in the case of formins) may be used to elicit observable phenotypes; this phenomenon is known as “synthetic lethality” or “synthetic toxicity” (see [168]). Indeed, seedling roots of *Arabidopsis* mutants lacking AtFH8 exhibited increased sensitivity towards the actin-depolymerizing drug LatB compared to wild-type plants [169].

Besides the already mentioned tobacco and moss studies [163, 166], there is not much data on Class I formins from other plant species. A tomato member of this family has been recently found as an interacting partner of a resistance protein involved in the response to fungal pathogens; however, silencing of the formin did not influence pathogen sensitivity [170], somewhat reminiscent of the already mentioned case of AtFH6 whose mutation did not affect nematode response, despite massive expression in nematode-induced galls [159].

Compared to Class I formins, even less is known about their Class II counterparts; this may be partly due to the combination of usually low expression levels and complex locus structures that make prediction of cDNA sequences and PCR-based cloning difficult. Actin nucleation and bundling have been demonstrated for rice FH5, a canonical Class II formin, which can also bind microtubules and whose mutation exhibits a pleiotropic phenotype including stunted growth and organ bending, suggestive of problems with cell expansion and/or phytohormone transport [122, 123]. *Arabidopsis* AtFH14 was also found to bind both actin and microtubules, and its loss led to mild defects in cell division and meiosis [121]. In the absence of GOE-related motifs, the mechanism of microtubule-binding must necessarily be different from that found in Class I formins. Very recently, another *Arabidopsis* Class II formin, AtFH19, was reported to nucleate actin *in vitro*. Remarkably, it competes with AtFH1 in barbed-end binding and exhibits different kinetic properties, suggesting intriguing possibilities for modulation of actin dynamics by intreaction of Class I and Class II formins [171].

**Figure 3:**
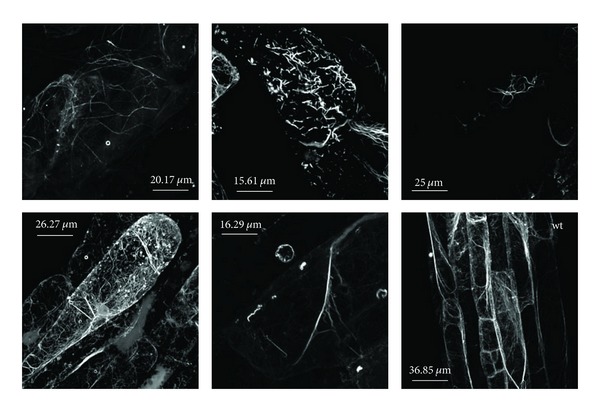
Confocal microscopy images of fragmented and malformed cortical actin cytoskeleton observed in the roots of 4-to-7-days-old *Arabidopsis thaliana* seedlings expressing the moderately toxic actin marker, GFP-tagged mouse talin, in a genetic background lacking the Class II outlier formin AtFH12. Corresponding tissue from a wild-type (wt) isogenic seedling is shown in the right bottom corner. Modified from [138]; confocal images obtained by the author.

The hypothesis that PTEN domains of typical Class II formins mediate binding to membranes (see above) has been recently confirmed for a *Physcomitrella patens* Class II formin [172]. Simultaneous RNAi knockdown of all moss Class II formins revealed that they are required for tip growth [166]. In angiosperms, phenotypes of Class II mutants are subtler. Besides the above-mentioned *Arabidopsis *and rice examples, loss of the outlier atypical *Arabidopsis* Class II formin AtFH12, which lacks the PTEN domain, caused only a minor decrease in the sensitivity of root growth towards LatB, raising the intriguing possibility that the PTEN-less Class II formin may somehow attenuate actin polymerization or destabilize actin filaments. Consistent with this hypothesis, mutants lacking AtFH12 also exhibited partial synthetic lethality with the fluorescent actin marker GFP-tagged mouse talin (GFP-mTalin), which is known to stabilize actin and induce actin-bundling, another example of the “synthetic lethality” phenomenon. Indeed, surviving plants carrying GFP-mTalin in the mutant background rapidly silenced the toxic transgene, and exhibited excessive and anomalous actin bundling in the still-expressing tissues ([139]; Figure 3).

Several Class II formins appear to respond to environmental cues; AtFH12 is induced by salt stress, but its loss does not affect salt sensitivity [139]. Although there are no data on the involvement of *Arabidopsis* Class II members in pathogen response, a typical representative of this clade was among genes dramatically repressed in phytoplasma-infected, diseased *Citrus aurantifolia* [173], suggesting that both Class I and Class II formins might be involved in the response to pathogens.

The extent of our current understanding concerning plant formins is therefore far behind the thesaurus of knowledge available on formins of fungi and metazoa, reflecting clearly the more than dozen years delay. Plant formin research has now reached the phase when any new observations spawn a host of questions to be addressed. Undoubtedly, major advances can be expected with the progress of *in vivo* imaging techniques such as the VAEM microscopy, allowing observations of individual microtubules or microfilaments *in vivo*, that has only recently been applied in first plant studies [174].

## 5. Conclusions

The progress in plant formin studies has been the topic, and mostly main focus, of several reviews in the last decade [104, 137, 175, 176]; however, these were either brief updates restricted by journal space, or focused on specific aspects of formin biology or biochemistry. Here I attempted to provide an exhaustive account of published work on plant formins in the broader context of current understanding of FH2 protein roles in general, and of the structure and function of the plant cell cortex. 

Although phenotypes of plant formin mutants are rarely dramatic due to the degeneracy of the extensive plant formin families and resulting mutual replaceability of products of multiple genes, the emerging picture suggests that FH2 proteins participate in multiple cellular processes crucial especially for precise control of cell morphogenesis, including various modes of cell expansion. 

Remarkably, formins appear to be not only important regulators of the actin cytoskeleton, but also prime candidates for mediating the co-ordination between microfilaments and microtubules also in plants, despite using mechanisms of microtubule interaction different from those of their metazoan counterparts.

The unique plant-specific domain structure of plant Class I formins enables these proteins to act as direct membrane-crossing linkers between the cytoskeleton in the cortical cytoplasm and the cell wall; however, this does not exclude the possibility that formins may also associate with intracellular membrane compartments such as the ER.

In summary, it becomes increasingly clear that plant formins are far from copying the tasks of their opisthokont counterparts; on the contrary, they operate in plant-specific contexts to accomplish plant-specific function, documenting thereby the extreme versatility of evolutionarily ancient protein domains and domain combinations such as, for example, the FH1-FH2 tandem or the PTEN domain.

## References

[1] Mathur J (2004). Cell shape development in plants. *Trends in Plant Science*.

[2] Mathur J (2005). Conservation of boundary extension mechanisms between plants and animals. *Journal of Cell Biology*.

[3] Boutté Y, Crosnier MT, Carraro N, Traas J, Satiat-Jeunemaitre B (2006). The plasma membrane recycling pathway and cell polarity in plants: studies on PIN proteins. *Journal of Cell Science*.

[4] Geldner N, Friml J, Stierhof YD, Jürgens G, Palme K (2001). Auxin transport inhibitors block PIN1 cycling and vesicle trafficking. *Nature*.

[5] Petrášek J, Černá A, Schwarzerová K, Elčkner M, Morris DA, Zažímalová E (2003). Do phytotropins inhibit auxin efflux by impairing vesicle traffic?. *Plant Physiology*.

[6] Žárský V, Cvrčková F, Potocký M, Hála M (2009). Exocytosis and cell polarity in plants—exocyst and recycling domains: tansley review. *New Phytologist*.

[7] Wasteneys GO (2004). Progress in understanding the role of microtubules in plant cells. *Current Opinion in Plant Biology*.

[8] Takahashi H, Hirota K, Kawahara A, Hayakawa E, Inoue Y (2003). Randomization of cortical microtubules in root epidermal cells induces root hair initiation in lettuce (*Lactuca sativa* L.) seedlings. *Plant and Cell Physiology*.

[9] Sieberer BJ, Timmers ACJ, Lhuissier FGP, Emons AMC (2002). Endoplasmic microtubules configure the subapical cytoplasm and are required for fast growth of *Medicago truncatula* root hairs. *Plant Physiology*.

[10] Morejohn LC, Bureau TE, Molè-Bajer J, Bajer AS, Fosket DE (1987). Oryzalin, a dinitroaniline herbicide, binds to plant tubulin and inhibits microtubule polymerization in vitro. *Planta*.

[11] Sugimoto K, Himmelspach R, Williamson RE, Wasteneys GO (2003). Mutation or drug-dependent microtubule disruption causes radial swelling without altering parallel cellulose microfibril deposition in *Arabidopsis* root cells. *Plant Cell*.

[12] Baskin TI, Beemster GTS, Judy-March JE, Marga F (2004). Disorganization of cortical microtubules stimulates tangential expansion and reduces the uniformity of cellulose microfibril alignment among cells in the root of *Arabidopsis*. *Plant Physiology*.

[13] Lindeboom J, Mulder BM, Vos JW, Ketelaar T, Emons AMC (2008). Cellulose microfibril deposition: coordinated activity at the plant plasma membrane. *Journal of Microscopy*.

[14] Sieberer BJ, Ketelaar T, Esseling JJ, Emons AMC (2005). Microtubules guide root hair tip growth. *New Phytologist*.

[15] Bao Y, Kost B, Chua NH (2001). Reduced expression of *α*-tubulin genes in *Arabidopsis thaliana* specifically affects root growth and morphology, root hair development and root gravitropism. *Plant Journal*.

[16] Gossot O, Geitmann A (2007). Pollen tube growth: coping with mechanical obstacles involves the cytoskeleton. *Planta*.

[17] Potocký M, Eliáš M, Profotová B, Novotná Z, Valentová O, Žárský V (2003). Phosphatidic acid produced by phospholipase D is required for tobacco pollen tube growth. *Planta*.

[18] Huang S, An YQ, McDowell JM, McKinney EC, Meagher RB (1997). The *Arabidopsis* ACT11 actin gene is strongly expressed in tissues of the emerging inflorescence, pollen, and developing ovules. *Plant Molecular Biology*.

[19] Gilliland LU, Kandasamy MK, Pawloski LC, Meagher RB (2002). Both vegetative and reproductive actin isovariants complement the stunted root hair phenotype of the *Arabidopsis act2-1* mutation. *Plant Physiology*.

[20] Pawloski LC, Kandasamy MK, Meagher RB (2006). The late pollen actins are essential for normal male and female development in *Arabidopsis*. *Plant Molecular Biology*.

[21] Cvrčková F, Bezvoda R, Žárský V (2010). Computational identification of root hair-specific genes in *Arabidopsis*. *Plant Signaling and Behavior*.

[22] Kandasamy MK, Gilliland LU, McKinney EC, Meagher RB (2001). One plant actin isovariant, ACT7, is induced by auxin and required for normal callus formation. *Plant Cell*.

[23] Gilliland LU, Pawloski LC, Kandasamy MK, Meagher RB (2003). *Arabidopsis* actin gene *ACT7* plays an essential role in germination and root growth. *Plant Journal*.

[24] Bannigan A, Wiedemeier AMD, Williamson RE, Overall RL, Baskin TI (2006). Cortical microtubule arrays lose uniform alignment between cells and are oryzalin resistant in the *Arabidopsis* mutant, radially swollen 6. *Plant and Cell Physiology*.

[25] Collings DA, Lill AW, Himmelspach R, Wasteneys GO (2006). Hypersensitivity to cytoskeletal antagonists demonstrates microtubule—microfilament cross-talk in the control of root elongation in *Arabidopsis thaliana*. *New Phytologist*.

[26] Schwab B, Mathur J, Saedler R (2003). Regulation of cell expansion by the DISTORTED genes in *Arabidopsis thaliana*: actin controls the spatial organization of microtubules. *Molecular Genetics and Genomics*.

[27] Saedler R, Mathur N, Srinivas BP, Kernebeck B, Hülskamp M, Mathur J (2004). Actin control over microtubules suggested by DISTORTED2 encoding the *Arabidopsis* ARPC2 subunit homolog. *Plant and Cell Physiology*.

[28] Li S, Blanchoin L, Yang Z, Lord EM (2003). The putative *Arabidopsis* Arp2/3 complex controls leaf cell morphogenesis. *Plant Physiology*.

[29] Le J, El-Assal SED, Basu D, Saad ME, Szymanski DB (2003). Requirements for *Arabidopsis* ATARP2 and ATARP3 during epidermal development. *Current Biology*.

[30] Mathur J, Mathur N, Kirik V, Kernebeck B, Srinivas BP, Hülskamp M (2003). *Arabidopsis* crooked encodes for the smallest subunit of the ARP2/3 complex and controls cell shape by region specific fine F-actin formation. *Development*.

[31] Mathur J, Mathur N, Kernebeck B, Hülskamp M (2003). Mutations in actin-related proteins 2 and 3 affect cell shape development in *Arabidopsis*. *Plant Cell*.

[32] Perroud PF, Quatrano RS (2006). The role of ARPC4 in tip growth and alignment of the polar axis in filaments of *Physcomitrella patens*. *Cell Motility and the Cytoskeleton*.

[33] Finka A, Saidi Y, Goloubinoff P, Neuhaus JM, Zrÿd JP, Schaefer DG (2008). The knock-out of ARP3a gene affects F-actin cytoskeleton organization altering cellular tip growth, morphology and development in moss *Physcomitrella patens*. *Cell Motility and the Cytoskeleton*.

[34] Fišerová J, Schwarzerová K, Petrášek J, Opatrný Z (2006). ARP2 and ARP3 are localized to sites of actin filament nucleation in tobacco BY-2 cells. *Protoplasma*.

[35] Baluška F, Salaj J, Mathur J (2000). Root hair formation: F-actin-dependent tip growth is initiated by local assembly of profilin-supported F-actin meshworks accumulated within expansin-enriched bulges. *Developmental Biology*.

[36] Ringli C, Baumberger N, Diet A, Frey B, Keller B (2002). *ACTIN2* is essential for bulge site selection and tip growth during root hair development of *Arabidopsis*. *Plant Physiology*.

[37] Ketelaar T, De Ruijter NCA, Emons AMC (2003). Unstable F-actin specifies the area and microtubule direction of cell expansion in *Arabidopsis* root hairs. *Plant Cell*.

[38] Carol RJ, Dolan L (2002). Building a hair: tip growth in *Arabidopsis thaliana* root hairs. *Philosophical Transactions of the Royal Society B*.

[39] Ojangu EL, Järve K, Paves H, Truve E (2007). *Arabidopsis thaliana* myosin XIK is involved in root hair as well as trichome morphogenesis on stems and leaves. *Protoplasma*.

[40] Vidali L, McKenna ST, Hepler PK (2001). Actin polymerization is essential for pollen tube growth. *Molecular Biology of the Cell*.

[41] Li Y, Zee SY, Liu YM, Huang BQ, Yen LF (2001). Circular F-actin bundles and a G-actin gradient in pollen and pollen tubes of *Lilium davidii*. *Planta*.

[42] Lovy-Wheeler A, Wilsen KL, Baskin TI, Hepler PK (2005). Enhanced fixation reveals the apical cortical fringe of actin filaments as a consistent feature of the pollen tube. *Planta*.

[43] Lenartowska M, Michalska A (2008). Actin filament organization and polarity in pollen tubes revealed by myosin II subfragment 1 decoration. *Planta*.

[44] Gibbon BC, Kovar DR, Staiger CJ (1999). Latrunculin B has different effects on pollen germination and tube growth. *Plant Cell*.

[45] Chen T, Teng N, Wu X (2007). Disruption of actin filaments by latrunculin B affects cell wall construction in *Picea meyeri* pollen tube by disturbing vesicle trafficking. *Plant and Cell Physiology*.

[46] Cárdenas L, Lovy-Wheeler A, Wilsen KL, Hepler PK (2005). Actin polymerization promotes the reversal of streaming in the apex of pollen tubes. *Cell Motility and the Cytoskeleton*.

[47] McFarlane HE, Young RE, Wasteneys GO, Samuels AL (2008). Cortical microtubules mark the mucilage secretion domain of the plasma membrane in *Arabidopsis* seed coat cells. *Planta*.

[48] Kleine-Vehn J, Dhonukshe P, Swarup R, Bennett M, Friml J (2006). Subcellular trafficking of the *Arabidopsis* auxin influx carrier AUX1 uses a novel pathway distinct from PIN1. *Plant Cell*.

[49] Maisch J, Nick P (2007). Actin is involved in auxin-dependent patterning. *Plant Physiology*.

[50] Dhonukshe P, Mathur J, Hülskamp M, Gadella TWJ (2005). Microtubule plus-ends reveal essential links between intracellular polarization and localized modulation of endocytosis during division-plane establishment in plant cells. *BMC Biology*.

[51] Karahara I, Suda J, Tahara H (2009). The preprophase band is a localized center of clathrin-mediated endocytosis in late prophase cells of the onion cotyledon epidermis. *Plant Journal*.

[52] Twell D, Park SK, Hawkins TJ (2002). MORI/GEM1 has an essential role in the plant-specific cytokinetic phragmoplast. *Nature Cell Biology*.

[53] Eleftheriou EP, Baskin TI, Hepler PK (2005). Aberrant cell plate formation in the *Arabidopsis thaliana* microtubule organization 1 mutant. *Plant and Cell Physiology*.

[54] Kawamura E, Himmelspach R, Rashbrooke MC (2006). MICROTUBULE ORGANIZATION 1 regulates structure and function of microtubule arrays during mitosis and cytokinesis in the *Arabidopsis* root. *Plant Physiology*.

[55] Hepler PK, Valster A, Molchan T, Vos JW (2002). Roles for Kinesin and myosin during cytokinesis. *Philosophical Transactions of the Royal Society B*.

[56] Seguí-Simarro JM, Austin JR, White EA, Staehelin LA (2004). Electron tomographic analysis of somatic cell plate formation in meristematic cells of *Arabidopsis* preserved by high-pressure freezing. *Plant Cell*.

[57] Higaki T, Kutsuna N, Sano T, Hasezawa S (2008). Quantitative analysis of changes in actin microfilament contribution to cell plate development in plant cytokinesis. *BMC Plant Biology*.

[58] Žárský V, Potocký M (2010). Recycling domains in plant cell morphogenesis: small GTPase effectors, plasma membrane signalling and the exocyst. *Biochemical Society Transactions*.

[59] Žárský V, Fowler JE, Emons AMC, Ketelaar T (2009). ROP, (Rho-related protein from plants) GTPases for spatial control of root hair morphogenesis. *Root Hairs*.

[60] Cvrčková F, Rivero F, Bavlnka B (2004). Evolutionarily conserved modules in actin nucleation: lessons from *Dictyostelium discoideum* and plants. *Protoplasma*.

[61] Rivero F, Cvrčková F, Jekely G (2007). Origins and evolution of the actin cytoskeleton. *Eukaryotic Membranes and Cytoskeleton: Origins and Evolution*.

[62] Wasserman S (1998). FH proteins as cytoskeletal organizers. *Trends in Cell Biology*.

[63] Zigmond SH (2004). Formin-induced nucleation of actin filaments. *Current Opinion in Cell Biology*.

[64] Faix J, Grosse R (2006). Staying in shape with formins. *Developmental Cell*.

[65] Young KG, Copeland JW (2010). Formins in cell signaling. *Biochimica et Biophysica Acta*.

[66] Liu R, Linardopoulou EV, Osborn GE, Parkhurst SM (2010). Formins in development: orchestrating body plan origami. *Biochimica et Biophysica Acta*.

[67] Woychik RP, Stewart TA, Davis LG (1985). An inherited limb deformity created by insertional mutagenesis in a transgenic mouse. *Nature*.

[68] De la Pompa JL, James D, Zeller R (1995). Limb deformity proteins during avian neurulation and sense organ development. *Developmental Dynamics*.

[69] Trumpp A, Blundell PA, De la Pompa JL, Zeller R (1992). The chicken limb deformity gene encodes nuclear proteins expressed in specific cell types during morphogenesis. *Genes and Development*.

[70] Zeller R, Haramis AG, Zuniga A (1999). Formin defines a large family of morphoregulatory genes and functions in establishment of the polarising region. *Cell and Tissue Research*.

[71] Wang CC, Chan DC, Leder P (1997). The mouse formin (Fmn) gene: genomic structure, novel exons, and genetic mapping. *Genomics*.

[72] Evangelista M, Blundell K, Longtine MS (1997). Bni1p, a yeast formin linking Cdc42p and the actin cytoskeleton during polarized morphogenesis. *Science*.

[73] Kamei T, Tanaka K, Hihara T (1998). Interaction of Bnr1p with a novel Src homology 3 domain-containing Hof1p: implication in cytokinesis in *Saccharomyces cerevisiae*. *Journal of Biological Chemistry*.

[74] Lee L, Klee SK, Evangelista M, Boone C, Pellman D (1999). Control of mitotic spindle position by the *Saccharomyces cerevisiae* formin Bni1p. *Journal of Cell Biology*.

[75] Van Reenen CG, Meuwissen THE, Hopster H, Oldenbroek K, Kruip TAM, Blokhuis HJ (2001). Transgenesis may affect farm animal welfare: a case for systematic risk assessment. *Journal of Animal Science*.

[76] Zuniga A, Michos O, Spitz F (2004). Mouse limb deformity mutations disrupt a global control region within the large regulatory landscape required for Gremlin expression. *Genes and Development*.

[77] Zhou F, Leder P, Zuniga A, Dettenhofer M (2009). Formin1 disruption confers oligodactylism and alters Bmp signaling. *Human Molecular Genetics*.

[78] Emmons S, Phan H, Calley J, Chen W, James B, Manseau L (1995). *cappuccino*, a *Drosophila* maternal effect gene required for polarity of the egg and embryo, is related to the vertebrate limb deformity locus. *Genes and Development*.

[79] Castrillon DH, Wasserman SA (1994). *diaphanous* is required for cytokinesis in *Drosophila* and shares domains of similarity with the products of the limb deformity gene. *Development*.

[80] Vogt TF, Jackson-Grusby L, Rush J, Leder P (1993). Formins: phosphoprotein isoforms encoded by the mouse limb deformity locus. *Proceedings of the National Academy of Sciences of the United States of America*.

[81] Chan DC, Leder P (1996). Genetic evidence that formins function within the nucleus. *Journal of Biological Chemistry*.

[82] Haramis AG, Brown JM, Zeller R (1995). The limb deformity mutation disrupts the SHH/FGF-4 feedback loop and regulation of 5′ HoxD genes during limb pattern formation. *Development*.

[83] Higgs HN, Peterson KJ (2005). Phylogenetic analysis of the formin homology 2 domain. *Molecular Biology of the Cell*.

[84] Grunt M, Žárský V, Cvrčková F (2008). Roots of angiosperm formins: the evolutionary history of plant FH2 domain-containing proteins. *BMC Evolutionary Biology*.

[85] Chalkia D, Nikolaidis N, Makalowski W, Klein J, Nei M (2008). Origins and evolution of the formin multigene family that is involved in the formation of actin filaments. *Molecular Biology and Evolution*.

[86] Rivero F, Muramoto T, Meyer AK, Urushihara H, Uyeda TQP, Kitayama C (2005). A comparative sequence analysis reveals a common GBD/FH3-FH1-FH2-DAD architecture in formins from *Dictyostelium*, fungi and metazoa. *BMC Genomics*.

[87] Petersen J, Nielsen O, Egel R, Hagan IM (1998). FH3, a domain found in formins, targets the fission yeast formin FUS1 to the projection tip during conjugation. *Journal of Cell Biology*.

[88] Watanabe N, Madaule P, Reid T (1997). p140mDia, a mammalian homolog of *Drosophila diaphanous*, is a target protein for Rho small GTPase and is a ligand for profilin. *EMBO Journal*.

[89] Tominaga T, Sahai E, Chardin P, McCormick F, Courtneidge SA, Alberts AS (2000). Diaphanous-related formins bridge Rho GTPase and Src tyrosine kinase signaling. *Molecular Cell*.

[90] Alberts AS (2002). Diaphanous-related Formin homology proteins. *Current Biology*.

[91] Ridley AJ (2006). Rho GTPases and actin dynamics in membrane protrusions and vesicle trafficking. *Trends in Cell Biology*.

[92] Hall A (2005). Rho GTPases and the control of cell behaviour. *Biochemical Society Transactions*.

[93] Sorokina EM, Chernoff J (2005). Rho-GTPases: new members, new pathways. *Journal of Cellular Biochemistry*.

[94] Mucha E, Fricke I, Schaefer A, Wittinghofer A, Berken A (2011). Rho proteins of plants - Functional cycle and regulation of cytoskeletal dynamics. *European Journal of Cell Biology*.

[95] Kato T, Watanabe N, Morishima Y, Fujita A, Ishizaki T, Narumiya S (2001). Localization of a mammalian homolog of *diaphanous*, mDia1, to the mitotic spindle in HeLa cells. *Journal of Cell Science*.

[96] Ishizaki T, Morishima Y, Okamoto M, Furuyashiki T, Kato T, Narumiya S (2001). Coordination of microtubules and the actin cytoskeleton by the Rho effector mDia1. *Nature Cell Biology*.

[97] Higgs HN (2005). Formin proteins: a domain-based approach. *Trends in Biochemical Sciences*.

[98] Young KG, Thurston SF, Copeland S, Smallwood C, Copeland JW (2008). INF1 is a novel microtubule-associated formin. *Molecular Biology of the Cell*.

[99] Sagot I, Klee SK, Pellman D (2002). Yeast formins regulate cell polarity by controlling the assembly of actin cables. *Nature Cell Biology*.

[100] Sagot I, Rodal AA, Moseley J, Goode BL, Pellman D (2002). An actin nucleation mechanism mediated by Bni1 and profilin. *Nature Cell Biology*.

[101] Xu Y, Moseley JB, Sagot I (2004). Crystal structures of a formin homology-2 domain reveal a tethered dimer architecture. *Cell*.

[102] Goode BL, Eck MJ (2007). Mechanism and function of formins in the control of actin assembly. *Annual Review of Biochemistry*.

[103] Paul AS, Pollard TD (2009). Review of the mechanism of processive actin filament elongation by formins. *Cell Motility and the Cytoskeleton*.

[104] Blanchoin L, Staiger CJ (2010). Plant formins: diverse isoforms and unique molecular mechanism. *Biochimica et Biophysica Acta*.

[105] Kovar DR, Kuhn JR, Tichy AL, Pollard TD (2003). The fission yeast cytokinesis formin Cdc12p is a barbed end actin filament capping protein gated by profilin. *Journal of Cell Biology*.

[106] Copeland SJ, Green BJ, Burchat S, Papalia GA, Banner D, Copeland JW (2007). The diaphanous inhibitory domain/diaphanous autoregulatory domain interaction is able to mediate heterodimerization between mDia1 and mDia2. *Journal of Biological Chemistry*.

[107] Sun H, Schlondorff JS, Brown EJ, Higgs HN, Pollak MR (2011). Rho activation of mDia formins is modulated by an interaction with inverted formin 2 (INF2). *Proceedings of the National Academy of Sciences of the United States of America*.

[108] Kerkhoff E (2011). Actin dynamics at intracellular membranes: the Spir/formin nucleator complex. *European Journal of Cell Biology*.

[109] Brandt DT, Grosse R (2007). Get to grips: steering local actin dynamics with IQGAPs. *EMBO Reports*.

[110] Liu R, Abreu-Blanco MT, Barry KC, Linardopoulou EV, Osborn GE, Parkhurst SM (2009). Wash functions downstream of Rho and links linear and branched actin nucleation factors. *Development*.

[111] Michelot A, Guérin C, Huang S (2005). The formin homology 1 domain modulates the actin nucleation and bundling activity of *Arabidopsis* FORMIN1. *Plant Cell*.

[112] Michelot A, Derivery E, Paterski-Boujemaa R (2006). A novel mechanism for the formation of actin-filament bundles by a
nonprocessive formin. *Current Biology*.

[113] Machaidze G, Sokoll A, Shimada A (2010). Actin filament bundling and different nucleating effects of mouse
diaphanous-related formin Fh2 domains on actin/adf and actin/cofilin complexes. *Journal of Molecular Biology*.

[114] Harris ES, Rouiller I, Hanein D, Higgs HN (2006). Mechanistic differences in actin bundling activity of two mammalian formins, FRL1 and mDia2. *Journal of Biological Chemistry*.

[115] Esue O, Harris ES, Higgs HN, Wirtz D (2008). The filamentous actin cross-linking/bundling activity of mammalian
formins. *Journal of Molecular Biology*.

[116] Delgehyr N, Lopes CSJ, Moir CA, Huisman SM, Segal M (2008). Dissecting the involvement of formins in Bud6p-mediated cortical capture of microtubules in *S. cerevisiae*. *Journal of Cell Science*.

[117] Bartolini F, Gundersen GG (2010). Formins and microtubules. *Biochimica et Biophysica Acta*.

[118] Bartolini F, Moseley JB, Schmoranzer J, Cassimeris L, Goode BL, Gundersen GG (2008). The formin mDia2 stabilizes microtubules independently of its actin nucleation activity. *Journal of Cell Biology*.

[119] Mao Y (2011). FORMIN a link between kinetochores and microtubule ends. *Trends in Cell Biology*.

[120] Deeks MJ, Fendrych M, Smertenko A (2010). The plant formin AtFH4 interacts with both actin and microtubules, and contains a newly identified microtubule-binding domain. *Journal of Cell Science*.

[121] Li Y, Shen Y, Cai C (2010). The type II *Arabidopsis* formin14 interacts with microtubules and microfilaments to regulate cell division. *Plant Cell*.

[122] Zhang Z, Zhang Y, Tan H (2011). *RICE MORPHOLOGY DETERMINANT* encodes the type II formin FH_5_ and regulates rice morphogenesis. *Plant Cell*.

[123] Yang W, Ren S, Zhang X (2011). BENT UPPERMOST INTERNODE1 Encodes the class II formin FH5 crucial for actin organization and rice development. *Plant Cell*.

[124] Kanaya H, Takeya R, Takeuchi K, Watanabe N, Jing N, Sumimoto H (2005). Fhos2, a novel formin-related actin-organizing protein, probably associates with the nestin intermediate filament. *Genes to Cells*.

[125] Johnston RJ, Copeland JW, Fasnacht M (2006). An unusual Zn-finger/FH2 domain protein controls a left/right asymmetric neuronal fate decision in *C. elegans*. *Development*.

[126] Bedford MT, Reed R, Leder P (1998). WW domain-mediated interactions reveal a spliceosome-associated protein that binds a third class of proline-rich motif: the proline glycine and methionine-rich motif. *Proceedings of the National Academy of Sciences of the United States of America*.

[127] Fujiwara T, Tanaka K, Mino A (1998). Rho1p-Bni1p-Spa2p interactions: implication in localization of Bni1p at the bud site and regulation of the actin cytoskeleton in *Saccharomyces cerevisiae*. *Molecular Biology of the Cell*.

[128] Afshar K, Stuart B, Wasserman SA (2000). Functional analysis of the *Drosophila diaphanous* FH protein in early embryonic development. *Development*.

[129] Severson AF, Baillie DL, Bowerman B (2002). A Formin Homology protein and a profilin are required for cytokinesis and Arp2/3-independent assembly of cortical microfilaments in *C. elegans*. *Current Biology*.

[130] Gill MB, Roecklein-Canfield J, Sage DR (2004). EBV attachment stimulates FHOS/FHOD1 redistribution and co-aggregation with CD21: formin interactions with the cytoplasmic domain of human CD21. *Journal of Cell Science*.

[131] Carramusa L, Ballestrem C, Zilberman Y, Bershadsky AD (2007). Mammalian diaphanous-related formin Dia1 controls the organization of E-cadherin-mediated cell-cell junctions. *Journal of Cell Science*.

[132] Mellor H (2010). The role of formins in filopodia formation. *Biochimica et Biophysica Acta*.

[133] Yang C, Svitkina T (2011). Filopodia initiation: focus on the Arp2/3 complex and formins. *Cell Adhesion & Migration*.

[134] Banno H, Chua NH (2000). Characterization of the *Arabidopsis* formin-like protein AFH1 and its interacting protein. *Plant and Cell Physiology*.

[135] Cvrčková F (2000). Are plant formins integral membrane proteins?. *Genome Biology*.

[136] Kaul S, Koo HL, Jenkins J (2000). Analysis of the genome sequence of the flowering plant *Arabidopsis thaliana*. *Nature*.

[137] Deeks MJ, Hussey PJ, Davies B (2002). Formins: intermediates in signal-transduction cascades that affect cytoskeletal reorganization. *Trends in Plant Science*.

[138] Cvrčková F, Novotný M, Pícková D, Žárský V (2004). Formin homology 2 domains occur in multiple contexts in angiosperms. *BMC Genomics*.

[139] Cvrčková F, Grunt M, Žárský V (2012). Expression of GFP-mTalin reveals an actin-related role for the *Arabidopsis* Class II formin AtFH12. *Biologia Plantarum*.

[140] Kieliszewski MJ, Lamport DTA (1994). Extensin: repetitive motifs, functional sites, post-translational codes, and phylogeny. *Plant Journal*.

[141] Li J, Yen C, Liaw D (1997). *PTEN*, a putative protein tyrosine phosphatase gene mutated in human brain, breast, and prostate cancer. *Science*.

[142] Steck PA, Pershouse MA, Jasser SA (1997). Identification of a candidate tumour suppressor gene, MMAC1, at chromosome 10q23.3 that is mutated in multiple advanced cancers. *Nature Genetics*.

[143] Li L, Ernsting BR, Wishart MJ, Lohse DL, Dixon JE (1997). A family of putative tumor suppressors is structurally and functionally conserved in humans and yeast. *Journal of Biological Chemistry*.

[144] von Stein W, Ramrath A, Grimm A, Müller-Borg M, Wodarz A (2005). Direct association of Bazooka/PAR-3 with the lipid phosphatase PTEN reveals a link between the PAR/aPKC complex and phosphoinositide signaling. *Development*.

[145] Gupta R, Ting JTL, Sokolov LN, Johnson SA, Luan S (2002). A tumor suppressor homolog, AtPTEN1, is essential for pollen development in *Arabidopsis*. *Plant Cell*.

[146] Cvrčková F, Grunt M, Bezvoda R (2012). Evolution of the land plant exocyst complexes. *Frontiers in Plant Science*.

[147] Hála M, Cole R, Synek L (2008). An exocyst complex functions in plant cell growth in *Arabidopsis* and tobacco. *Plant Cell*.

[148] Fendrych M, Žárský V, Synek L (2010). The *Arabidopsis* exocyst complex is involved in cytokinesis and cell plate maturation. *Plant Cell*.

[149] Kulich I, Cole R, Drdová E (2010). *Arabidopsis* exocyst subunits SEC8 and EXO70A1 and exocyst interactor ROH1 are involved in the localized deposition of seed coat pectin. *New Phytologist*.

[150] Brookfield JFY (1997). Genetic Redundancy. *Advances in Genetics*.

[151] Gibson TA, Goldberg DS (2009). Questioning the ubiquity of neofunctionalization. *PLoS Computational Biology*.

[152] Nasmyth K, Dirick L, Surana U, Amon A, Cvrčková F (1991). Some facts and thoughts on cell cycle control in yeast. *Cold Spring Harbor Symposia on Quantitative Biology*.

[153] Hruz T, Laule O, Szabo G (2008). Genevestigator v3: a reference expression database for the meta-analysis of transcriptomes. *Advances in Bioinformatics*.

[154] Mazzucotelli E, Belloni S, Marone D (2006). The E3 ubiquitin ligase gene family in plants: regulation by degradation. *Current Genomics*.

[155] Martinière A, Gayral P, Hawes C, Runions J (2011). Building bridges: formin1 of *Arabidopsis* forms a connection between the cell wall and the actin cytoskeleton. *Plant Journal*.

[156] Deeks MJ, Cvrčková F, Machesky LM (2005). *Arabidopsis* group Ie formins localize to specific cell membrane domains, interact with actin-binding proteins and cause defects in cell expansion upon aberrant expression. *New Phytologist*.

[157] Baluška F, Hlavačka A (2005). Plant formins come of age: something special about cross-walls. *New Phytologist*.

[158] Ingouff M, Fitz Gerald JN, Guérin C (2005). Plant formin *AtFH5* is an evolutionarily conserved actin nucleator involved in cytokinesis. *Nature Cell Biology*.

[159] Favery B, Chelysheva LA, Lebris M (2004). *Arabidopsis* formin AtFH6 is a plasma membrane-associated protein upregulated in giant cells induced by parasitic nematodes. *Plant Cell*.

[160] Van Damme D, Bouget FY, Van Poucke K, Inzé D, Geelen D (2004). Molecular dissection of plant cytokinesis and phragmoplast structure: a survey of GFP-tagged proteins. *Plant Journal*.

[161] Cheung AY, Wu HM (2004). Overexpression of an *Arabidopsis* formin stimulates supernumerary actin cable formation from Pollen tube cell membrane. *Plant Cell*.

[162] Yi K, Guo C, Chen D, Zhao B, Yang B, Ren H (2005). Cloning and functional characterization of a formin-like protein (AtFH8) from *Arabidopsis*. *Plant Physiology*.

[163] Ye J, Zheng Y, Yan A (2009). *Arabidopsis* formin3 directs the formation of actin cables and polarized growth in pollen tubes. *Plant Cell*.

[164] Cheung AY, Niroomand S, Zou Y, Wu HM (2010). A transmembrane formin nucleates subapical actin assembly and controls tip-focused growth in pollen tubes. *Proceedings of the National Academy of Sciences of the United States of America*.

[165] Mathur J (2005). The ARP2/3 complex: giving plant cells a leading edge. *BioEssays*.

[166] Vidali L, Van Gisbergen PAC, Guérin C (2009). Rapid formin-mediated actin-filament elongation is essential for polarized plant cell growth. *Proceedings of the National Academy of Sciences of the United States of America*.

[167] Gerald JNF, Hui PS, Berger F (2009). Polycomb group-dependent imprinting of the actin regulator *AtFH5* regulates morphogenesis in *Arabidopsis thaliana*. *Development*.

[168] Hartman JL, Garvik B, Hartwell L (2001). Cell Biology: principles for the buffering of genetic variation. *Science*.

[169] Xue XH, Guo CQ, Du F, Lu QL, Zhang CM, Ren HY (2011). AtFH8 is involved in root development under effect of low-dose latrunculin B in dividing cells. *Molecular Plant*.

[170] Lukasik-Shreepaathy E, Vossen JH, Tameking WIL, de Vroomen MJ, Cornelissen BJC, Takken FLW (2012). Protein-protein interactions as a proxy to monitor conformational changes and activation states of the tomato resistance protein I-2. *Journal of Experimental Botany*.

[171] Zheng Y, Xin H, Lin J, Liu -m C, Huang S (2012). An *Arabidopsis* class II formin, AtFH19, nucleates actin assembly, binds to the barbed end of actin filaments and antagonizes the effect of AtFH1 on actin dynamics. *Journal of Integrative Plant Biology*.

[172] van Gisbergen PA, Li M, Wu SZ, Bezanilla M (2012). Class II formin targeting to the cell cortex by binding PI(3, 5)P2 is essential for polarized growth. *The Journal of Cell Biology*.

[173] Zamharir MG, Mardi M, Alavi SM (2011). Identification of genes differentially expressed during interaction of Mexican lime tree infected with ‘*Candidatus Phytoplasma aurantifolia*’. *BMC Microbiology*.

[174] Wan Y, Ash WM3, Fan L, Hao H, Kim MK, Lin J (2011). Variable-angle total internal reflection fluorescence microscopy of intact cells of *Arabidopsis thaliana*. *Plant Methods*.

[175] Pei W, Du F, Zhang Y, He T, Ren H (2012). Control of the actin cytoskeleton in root hair development. *Plant Science*.

[176] Wang J, Xue X, Ren H (2012). New insights into the role of plant formins: regulating the organization of the actin and microtubule cytoskeleton. *Protoplasma*.

